# Socioeconomic differences in nicotine exposure and dependence in adult daily smokers

**DOI:** 10.1186/s12889-019-6694-4

**Published:** 2019-04-03

**Authors:** Allshine Chen, Michael Machiorlatti, Nicolle M. Krebs, Joshua E. Muscat

**Affiliations:** 1 Department of Biostatistics and Epidemiology, University of Oklahoma Health Sciences Center, 801 NE 13th Street CHB309, Box 26901, Oklahoma City, OK 73104 USA; 2 Department of Public Health Sciences, Penn State College of Medicine, 500 University Dr. CH69, Hershey, PA 17033 USA

**Keywords:** Tobacco use disorder, Income, Cotinine, Principal components analysis, Occupations

## Abstract

**Background:**

Socioeconomic status (SES) is a major determinant of tobacco use but little is known whether SES affects nicotine exposure and the degree of nicotine dependence.

**Methods:**

The Pennsylvania Adult Smoking Study is a cross-sectional study of smoke exposure and nicotine dependence among adults conducted in central Pennsylvania between June 2012 and April 2014. The study included several measures of SES, including assessments of education and household income, as well as occupation, home ownership, health insurance, household density and savings accounts. Measurements included saliva for the nicotine metabolites cotinine (COT), 3-‘hydroxycotinine (3HC) and total metabolites (COT +3HC). Puffing behavior was determined using portable smoking topography devices.

**Results:**

The income levels of lighter smokers (< 20 cigarettes per day) was $10,000 more than heavier smokers. Higher Fagerström Test for Nicotine Dependence scores were associated with lower income and job status, scores ranged from 5.4 in unemployed, 4.4 in blue-collar, and 3.8 in white-collar workers. In principal components analysis used to derive SES indicators, household income, number in household, and type of dwelling were the major SES correlates of the primary component. Job category was the major correlate of the second component. Lower SES predicted significantly higher adjusted total nicotine metabolite levels in the unemployed group. Job category was significantly associated with total daily puffs, with the highest level in the unemployed, followed by blue-collar workers, after adjustment for income.

**Conclusions:**

Among smokers, there was a relationship between lower SES and increased nicotine dependence, cigarettes per day and nicotine exposure, which varied by job type.

**Electronic supplementary material:**

The online version of this article (10.1186/s12889-019-6694-4) contains supplementary material, which is available to authorized users.

## Background

The role of disadvantaged social class in tobacco use is increasingly being recognized as a critical factor in tobacco use behaviors. In the recent National Cancer Institute Monograph 22 “A Socioecological Approach to Addressing Tobacco-Related Health Disparities,” it was noted that historically, individuals with higher incomes and levels of education were more likely to be smokers [1]. This has changed dramatically in the past few decades where smoking is now a habit that occurs predominantly in persons with a high school degree or less. Indicators of socioeconomic status (SES) such as levels of education and income show that low levels of educational achievement and poverty are major determinants of tobacco use and lower smoking cessation rates [2–6]. Yet another indicator of SES is occupation. As with education, tobacco use varies by occupation [4, 7], and is highest in mining, construction, vehicle mechanics and operations [8] [9]. Among the unemployed, smokers have longer-term unemployment and are paid less when obtaining jobs than unemployed non-smokers [10].

The reasons for these disparities may include early exposure to smoking, social pressure to smoke, lack of access or knowledge of inexpensive smoking cessation aids, stress, greater exposure to tobacco advertising and lack of efficacy of anti-tobacco messaging [11]. Smokers with low SES have higher smoking-related adverse health outcomes [4–6, 12–17]. Further, these health disparities have increased over the years [18]. While the need for smoking cessation in these individuals is a major public health goal, it has also been argued that decreasing smoking prevalence in these smokers should also be considered as a poverty reduction strategy [19, 20].

Collectively SES has been characterized in relation to tobacco use, age at initiation, and tobacco cessation. Individually, lower educational attainment has been shown to predict increasing number of cigarettes smoked in current smokers [21]^,^ [22]^,^ [23] and with years of smoking [24, 25]. Similar findings have been reported for income [24, 25].

However, very little is known whether SES affects levels of nicotine exposure and dependence in smokers. It is the nicotine in tobacco that is ultimately responsible for tobacco use being a leading cause of preventable mortality worldwide [26]. Lower education but not income was associated with a higher Fagerström Test for Nicotine Dependence score in current smokers of the Finnish FINRISK study [27]. Data from the National Health and Nutrition Examination Surveys (NHANES III and NHANES 1999–2000) and the Heath Survey for England (1993–1996, 1998, 2001) provide support that lower SES might increase nicotine exposure and dependence. The concentration of cotinine, the immediate metabolite of the addicting agent nicotine, was higher with lower individual educational attainment in both surveys, and with higher neighborhood deprivation in the NHANES surveys [28, 29]^,^ [30].

The current study’s main aim is to determine how SES affects levels of nicotine exposure and dependence. We hypothesized that lower SES (lower education and income) is associated with greater nicotine exposure and dependence. Additionally, since a wide range of SES variables beyond education and income were collected on each participant we used a principal components analysis to create a SES summarized variable.

## Methods

The Pennsylvania Adult Smoking Study (PASS) is a cross-sectional study of 352 adult cigarette smokers, completed in 14 counties of central Pennsylvania [31]. The sample size was based on effect sizes for cotinine between high and low SES groups, based on previous findings of sample means and standard deviations for cotinine [32]. A sample size of 280 yielded 80% power at an alpha of 0.05 for moderate effect sizes. Eligible subjects were ages 18–65 who currently smoked daily for at least one or more years. Subjects were recruited from June 2012 to April 2014, using primary recruitment methods reliant on internet and social media, radio advertisements, posted flyers, and word of mouth. Eligible participants gave written consent and attended two study visits, and upon completion, were provided with compensation. This study received approval from the Penn State Hershey College of Medicine Institutional Review Board (Hershey, Pennsylvania, USA).

### Data collection

Trained interviewers administered a multiple-domain, structured questionnaire to each subject during an in-home study visit. It contained questions on cigarette use history, demographic measures (e.g., age, gender, race, marital status), socioeconomic factors, smoking addiction items (quitting history, Fagerström Test for Nicotine Dependence [FTND]), stress measures, and medical history. To reduce potential bias, the study incorporated items from the Consensus Measures of Phenotypes and Exposures (PhenX) Toolkit version 5.1 (March 23, 2012), which are recommended consensus measures for attributes in biomedical science [33]. For example, household income assessments are based on a series of questions that narrows down the response to a range of categories that maximizes response rates. For education, there are 23 response categories that include every grade level, GED, and levels of higher education.

Participants were taught to use a smoking topography device (Smoking Puff Analyzer-Mobile [SPA-M], SODIM SAS, Fleury-les-Aubrais, France) and were given the device on the first study visit to use over a 2-day period in conjunction with all of their cigarettes smoked in that period. More details on the data collection and cleaning of the topography data are presented elsewhere [31]. The interviewer scheduled a second, follow-up visit to collect the SPA-M machine. The SPA-M software determines the puff flow (ml/s), the number of puffs, puff duration (s), the interval between puffs (s), and puff volume (ml). We calculated summarized variables, total daily puff volume and total daily puffs, from the topography data from a 24-h period.

### Biomarkers

Subjects gave saliva samples using SalivaBio Oral Swabs (Salimetrics, State College, Pennsylvania), which were analyzed using mass spectrometry for tobacco nicotine metabolites described elsewhere [31]. These included cotinine (COT) and 3’hydroxycotinine (3HC). Total salivary nicotine metabolites (cotinine + 3’hydroxycotinine; TSNM) were calculated as the molar sum of the former measurements. TSNM is perhaps the best measure of nicotine exposure, since the metabolism of COT to 3HC is affected by gender, race, and other factors [34].

### Socioeconomic variables and creation of an SES index

The PASS survey SES variables included annual household income, county of residence, job type/employment status, educational attainment (subject, spouse, parents), housing type and ownership, health insurance type, and number of adults/children who reside at residence. These variables were chosen as they provide adequate coverage of variables that have been shown to be strong SES indicators [35–38]. We also created an adjusted household income variable by dividing household income by household size. For job type, white-collared workers included jobs as managerial, business & finance, computer/math, architectural engineering, legal, physical or life sciences, healthcare technician or healthcare support, arts and media, education, and community service. Blue-collar jobs were those in food preparation, protection services, building maintenance, non-managerial sales/services/office administration, construction, production, farming, maintenance & repair, and transportation. These categories were created with guidance from the Bureau of Labor Statistics job categories in the Standard Occupational Classification Manual [39]. Area-based data included median household county income, downloaded from U.S. Census data for the state of Pennsylvania.

In the field of health policy and utilization, principal components analysis has become a preferred method for aggregating different variables to derive a single measure of SES [40, 41]. In the current study, the socioeconomic factors collected were analyzed using principal component analysis (PCA). Before entering into the PCA, continuous variables were normalized (mean = 0, standard deviation = 1) and categorical variables were dichotomized [40]. For years of education, we modeled the variable as both binary (< 12 years vs > 12 years) and on a continuous 5-point scale (representing the categories Less than HS Graduate, HS Graduate/GED, Some College, Associate’s Degree, Bachelor’s Degree or Greater). PCA was then used to extract components and generate an SES index. We note that the first principal component is the strongest index in terms of least squares variability and can be considered as our generated SES index [40].

### Statistical analysis

SAS version 9.4 (SAS Institute, Cary, North Carolina) was used for all analyses. A total of 326 of the 352 participants had complete topography data, and were included in the current analysis. In addition to the above socioeconomic measures, we also created derived measures that may discriminate higher from lower SES subjects. An employment variable was created that was dichotomized into employed individuals and unemployed individuals. For the purposes of this study we combined students, retired, unemployed (temporarily and looking for work) and disabled into one grouping called ‘unemployed’. There were 2 retired persons in the unemployed grouping.

Differences in the levels of the individual socioeconomic factors were computed by job type by ANOVA and Chi-Square tests. Simple linear regression was first performed to measure the association of the SES principal component and each covariate with nicotine metabolites. All two-way interactions were explored between SES and each covariate and retained if *p*-value < 0.05. Additional models were performed that included SES covariates that did not comprise the SES component using backward selection techniques. The results of the initial models indicated effect modification from job type. Final multiple regression models are presented using the SES primary principal component, controlling for the potential confounders including gender, BMI, and FTND. These models are presented separately for white-collar, blue-collar and unemployed. Model diagnostics were performed to assess the validity of a linear model. 3HC and TSNM were log transformed to improve the linearity of the model whereas no transformation was indicated for COT. For all analyses Adj-R^2^ and *p*-value were used to assess the associations.

The topography data was analyzed using multiple linear regressions. The effect of income on total daily puffs was determined by modeling household income as a continuous variable. We also estimated its effect by dividing total annual household income by household size, a measure commonly used in census data. The effect of job type on total daily puff volume was modeled by indicator variables for blue-collar jobs and the unemployed, with white-collar jobs serving as the reference group.

## Results

### Measures of SES

Tables 1 and 2 show the continuous and categorical sociodemographic variables and smoking variables for all subjects, and stratified by job type. The average age of respondents was 37.6 years and 88% were classified as white race. The average household income of respondents was $54.7 k, with significant differences across job types (*p* < 0.001) with averages of $34.4 k, $54.4 k and $70.1 k respectively for those unemployed, blue-collar, and white-collar workers. Household income adjusted for household size was also associated with job type, with the highest levels found in white collar subjects and the lowest in unemployed subjects. Education levels also varied significantly across job type with lowest levels in the unemployed, intermediate levels in blue-collar, and highest in white-collar workers (*p* < .001).

**Table 1:** Continuous smoking and socioeconomic variables by job type, Pennsylvania Adult Smoking Study

Socioeconomic variables	Overall		Unemployed^a^ (*n* = 63)		Blue Collar (*n* = 177)		White Collar (*n* = 86)		*P*-value
	Mean	SD	Mean	SD	Mean	SD	Mean	SD	
Age (years)	37.6	11.50	39.0	12.25	35.8	11.29	40.1	10.81	0.008
Household Income ($)	54,677	38,642	34,362	25,009	54,370	38,916	70,128	39,606	<.001
BMI	28.7	7.10	30.3	8.03	28.5	6.95	27.9	6.56	0.107
Height (inches)	66.8	3.97	65.6	3.62	67.3	4.04	66.8	3.91	0.017
Weight (pounds)	182.8	48.85	185.4	50.91	184.1	49.1	178.4	47.07	0.610
Number in household	3.3	1.50	3.3	1.43	3.4	1.62	3.0	1.26	0.082
Adults	2.3	0.99	2.4	1.1	2.4	1.03	2.2	0.8	0.427
Children	1.0	1.09	0.9	0.98	1.1	1.19	0.8	0.93	0.143
Smoking variables									
Cigarettes per day	16.6	8.07	18.2	8.56	16.8	7.96	15.0	7.72	0.047
FTND score	4.4	2.31	5.1	2.38	4.4	2.21	3.8	2.34	0.005
3HC (ng/mL)	115.7	86.23	130.5	85.88	112.3	86.02	112.1	86.81	0.337
Cotinine (ng/mL)	292.2	161.96	266.8	139.78	297.7	160.47	299.0	179.07	0.392
Nicotine (ng/mL)	558.3	803.74	839.3	1042.79	534.6	803.88	401.4	504.1	0.004
TSNM (mol/L)	0.3	0.08	0.3	0.07	0.3	0.08	0.3	0.09	0.919
NMR	0.42	0.26	0.6	0.32	0.4	0.24	0.4	0.25	<.001
Cotinine/cigarette	20.6	15.66	17.1	11.91	20.1	12.66	24.3	21.87	0.019

^a^Includes adult students, disabled, and retired participants. Abbreviations: 3HC: 3′-hydroxycotinine. TSNM: Total Salivary Nicotine Metabolites. NMR: Nicotine Metabolite Ratio. BMI: Body mass index. FTND: Fagerström Test for Nicotine Dependence

**Table 2:** Categorical smoking and socioeconomic variables by job type, Pennsylvania Adult Smoking Study

		Overall		Unemployed^a^(n = 63)		Blue Collar (n = 177)		White Collar (n = 86)		*p*-value
Socioeconomic Variables	Group	n	%	n	%	n	%	n	%	
Sex	Male	139	42.6	14	22.2	94	53.1	31	36.0	<.001
	Female	187	57.4	49	77.8	83	46.9	55	64.0	
Race	White	287	88.0	58	92.1	153	86.4	76	88.4	0.495
	Non White	39	12.0	5	7.9	24	13.6	10	11.6	
Education Level	Less than HS Graduate	22	6.7	11	17.5	9	5.1	2	2.3	<.001
	HS Graduate/GED	142	43.6	26	41.3	93	52.5	23	26.7	
	Some College	77	23.6	14	22.2	38	21.5	25	29.1	
	Associate’s Degree	54	16.6	8	12.7	28	15.8	18	20.9	
	Bachelor’s Degree or Greater	31	9.5	4	6.3	9	5.1	18	20.9	
Education Level (binary)	Less than HS Graduate/GED	22	6.7	11	17.5	9	5.0	2	2.3	0.001
	HS Graduate/GED or Greater	304	93.3	52	82.5	168	95.0	84	97.7	
Marital Status	Married	223	68.4	45	71.4	129	72.9	49	57.0	0.029
	Not Married	103	31.6	18	28.6	48	27.1	37	43.0	
Type of Dwelling	House	80	24.7	24	38.1	40	22.7	16	18.8	0.018
	Apartment/Mobile Home	244	75.3	39	61.9	136	77.3	69	81.2	
Mother’s Education	Less than HS Graduate	48	14.8	12	19.4	25	14.1	11	12.8	0.298
	HS Graduate/GED	168	51.7	37	59.7	86	48.6	45	52.3	
	Some College	29	8.9	3	4.8	18	10.2	8	9.3	
	Associate’s Degree	32	9.8	4	6.5	19	10.7	9	10.5	
	Bachelor’s Degree or Greater	36	11.1	2	3.2	22	12.4	12	14.0	
	Don’t know	12	3.7	4	6.5	7	4.0	1	1.2	
Mother’s Education (Binary)	Less than HS Graduate/GED	68	20.9	56	90.3	136	76.8	65	75.6	0.052
	HS Graduate/GED or Greater	257	79.1	6	9.7	41	23.2	21	24.4	
Father’s Education	Less than HS Graduate	57	18.0	14	23.3	31	17.8	12	14.5	0.557
	HS Graduate/GED or Greater	146	46.1	27	45.0	82	47.1	37	44.6	
	Some College	24	7.6	4	6.7	12	6.9	8	9.6	
	Associate’s Degree	20	6.3	5	8.3	10	5.7	5	6.0	
	Bachelor’s Degree or Greater	38	12.0	3	5.0	20	11.5	15	18.1	
	Don’t know	32	10.1	7	11.7	19	10.9	6	7.2	
Father’s Education (Binary)	Less than HS Graduate/GED	58	18.3	52	86.7	144	82.8	63	75.9	0.225
	HS Graduate/GED or Greater	259	81.7	8	13.3	30	17.2	20	24.1	
Home Mortgage	No Mortgage	66	20.2	11	17.5	43	24.3	12	14.0	0.122
	Rent/Still Paying	260	79.8	52	82.5	134	75.7	74	86.0	
Median Household County Income	< 52 k	34	10.5	10	15.9	19	10.8	5	5.9	0.085
	> 52 k, < 55.2 k	153	47.2	22	34.9	83	47.2	48	56.5	
	> 55.2 k	137	42.3	31	49.2	74	42.0	32	37.6	
Savings Account	No	142	43.6	41	65.1	78	44.1	23	26.7	<.001
	Yes	184	56.4	22	34.9	99	55.9	63	73.3	
Smoking Variables										
Cigarettes per day	< 20 CPD	259	80.2	43	69.4	139	79.0	77	90.6	0.005
	≥20 CPD	64	19.8	19	30.6	37	21.0	8	9.4	
Menthol cigarette smoker	No	146	45.5	28	44.4	74	43.0	44	51.2	0.457
	Yes	175	54.5	35	55.6	98	57.0	42	48.8	

^a^Includes adult students, disabled, and retired participants. Abbreviations: CPD: Cigarettes per day, HS: high school, GED: general education diploma

### SES and cigarettes per day

Cigarettes per day was significantly associated with lower income levels (*p* = 0.04). The mean household income (adjusted for sex) was about $61,500 in participants who smoked less than one pack per day, compared to $52,100 in participants who smoked one or more packs per day. Cigarettes per day was highest in the unemployed subjects (*p* < 0.05).

### SES and the FTND

A higher degree of nicotine dependence as measured by the FTND score was associated with a lower household income (*p* = 0.003). The mean FTND score for all subjects was 4.4, but varied by job type with means of 5.1, 4.4, and 3.8 for those unemployed, blue-collar, and white-collar jobs respectively (*p* = 0.005).

### Principal components analysis

The first principal component obtained from the PCA (PC1) explained 37.3% of the variability in the SES variables. The 3 SES variables included household income, number in household and type of dwelling residence (house vs apartment/mobile home). Income and number in the household had the strongest loadings and highest correlations, with dwelling playing a less significant role. All of the top 3 factor loadings had *p*-values < 0.0001 with correlations from 0.52–0.81. The second principal component explained 20% of the variation, with job category having the strongest loading. Additional file 1 shows the eigenvalues of the covariance matrix for the derived factors and Additional file 2 shows the factor loadings. The variability in the data is explained predominantly by the first 2–3 factors.

In simple linear regression models of the PCA-derived SES measure (PC1) on salivary nicotine and its metabolites (COT, 3HC, and TSNM), the coefficients for SES were negative, indicating that as SES increases, the metabolite measures are decreasing without adjustment by any covariate.

We found that for each metabolite measure, there was a significant interaction between SES and job type (*p*-values < 0.05). Consequently, we conducted models of the SES PC1 and nicotine metabolites stratified by job type and adjusted for confounders. Results are shown in Table 3. Increasing SES was negatively associated (β = − 65.48; *p* = 0.004) with cotinine for unemployed subjects. Among subjects with blue and white-collar jobs, SES was not associated with cotinine levels. Other predictors of cotinine in blue-collar workers were age, male sex, lower BMI and FTND (all p-values< 0.05). Age and FTND were associated with cotinine in white-collar workers.

**Table 3:** Multiple linear regression models of nicotine metabolites by job type, Pennsylvania Adult Smoking Study

	Unemployed/Retired/ Student/Disabled				Blue Collar				White Collar			
	Est	95% CI		*P*-value	Est	95% CI		P-value	Est	95% CI		*P*-value
Nicotine metabolite												
Cotinine												
Intercept	146.65	−5.84	299.13	0.059	136.21	29.14	243.28	0.013	141.19	−67.92	350.29	0.183
SES PC1	−65.48	− 109.58	−21.38	0.004	13.76	−4.60	32.12	0.141	27.91	−7.87	63.69	0.124
Age	*2.73*	*−0.29*	*5.75*	0.075	3.18	1.31	5.05	0.001	3.64	0.02	7.27	0.049
BMI	−1.85	−6.01	2.30	0.374	−3.16	−6.07	−0.25	0.034	−4.10	−9.82	1.63	0.156
FTND score	6.96	−8.14	22.06	0.359	23.22	13.74	32.69	<.001	27.88	11.35	44.41	0.001
Sex (M vs F)	67.87	−9.70	145.43	0.085	57.57	16.61	98.52	0.006	46.24	−31.31	123.78	0.238
Adj R^2^	0.279				0.245				0.209			
n	57				170				77			
3HC^a^												
Intercept	7.88	2.97	12.80	0.002	7.33	4.66	1.00	<.001	1.58	−2.77	5.92	0.472
SES PC1	0.07	−1.20	1.34	0.915	*−0.45*	*−0.92*	*0.02*	*0.059*	0.80	0.05	1.55	0.037
Age	0.11	0.02	0.19	0.014	0.07	0.02	0.12	0.006	0.10	0.03	0.18	0.007
FTND score	0.09	−0.38	0.57	0.697	0.38	0.12	0.65	0.005	0.87	0.50	1.24	<.001
Adj R^2^	0.228				0.230				0.364			
n	56				168				78			
TSNM^a^												
Intercept	−1.87	−2.26	−1.49	<.001	−1.73	−1.97	− 1.49	<.001	−2.03	− 2.41	− 1.65	<.001
SES PC1	−0.12	−0.23	0.00	0.042	−0.01	− 0.05	0.03	0.604	0.07	0.01	0.14	0.027
Age	0.01	0.00	0.02	0.047	0.01	0.00	0.01	0.001	0.01	0.00	0.02	0.005
BMI	0.00	−0.01	0.01	0.868	−0.01	−0.02	0.00	0.010	0.00	−0.01	0.01	0.410
FTND score	0.02	−0.02	0.05	0.391	0.06	0.04	0.08	<.001	0.08	0.05	0.11	<.001
Sex (M vs F)	0.15	−0.06	0.35	0.152	0.11	0.02	0.20	0.018	0.04	−0.10	0.18	0.602
Adj R^2^	0.205				0.2890				0.406			
n	55				168				77			

Abbreviations: 3HC: 3′-hydroxycotinine, TSNM: Total Salivary Nicotine Metabolites, NMR: Nicotine Metabolite Ratio, CPD: cigarettes per day, BMI: Body mass index, FTND: Fagerström Test for Nicotine Dependence, SES PC1: Socioeconomic status primary principal component

^a^3HC and TSNM were log-transformed

Table 3 also shows the relationships with 3HC and TSNM. Increasing SES was associated with lower levels of TSNM in unemployed subjects (β = − 0.12 *p* = 0.042). SES was not associated with TSNM in blue-collar workers (β = − 0.01 *p* = 0.604). SES was associated with higher TSNM levels in white collar jobs (β = 0.07 *p* = 0.027). Significant covariates included age for all job types and FTND for subjects with white and blue-collar jobs (*p* < 0.001). Male sex (*p* = 0.018) was significantly associated with TSNM in blue collar jobs. In alternative models that substituted cigarettes per day for FTND, findings were similar.

### SES and topography

The mean number of total daily puffs was 135 (SD = 89.0) in unemployed subjects, 118 (SD = 76.3) in blue-collar subjects, and 94 (SD = 58.7) in white-collar subjects. Table 4 shows that unemployed and blue-collar subjects take more puffs (*p*= 0.004) compared to white-collar subjects throughout the day while controlling for adjusted household income. The results were similar using total daily puff volume as the outcome measure. In the regression analysis, adjusted household income was not significantly related to total daily puffs (Table 4). When stratified by job type, among white collar subjects, increasing income was significantly associated with increasing puff volume whereas there was no association in the other two job groups (*p* < 0.05).

**Table 4:** Regression analysis of total number of daily puffs by job type and adjusted household income

Variable	Estimate	StandardError	*P*-value
Adjusted Household Income^a^	0.0003	0.0003	0.292
Unemployed	45.2319	13.4137	0.001
Blue-Collar	25.3138	12.4567	0.043
White-Collar	Reference		

^a^Household income/Household Size

## Discussion

There is a vast literature on the role of SES and tobacco use, yet many aspects of this relationship remain underexplored. In the current study, lower SES was associated with a higher number of cigarettes smoked per day, which is consistent with previous studies. We also found that cigarettes smoked per day was highest among the unemployed. Little is known about the smoking habits of the unemployed. In the Framingham Heart Study Offspring Cohort, men but not women who smoked and became unemployed increased their daily cigarette consumption [42]. These data indicate that unemployment or the stressors and lack of support structures associated with unemployment has an effect on cigarette consumption independent of low income.

SES is a construct that measures the concept of social position or class, or the ability to obtain desired resources such as material goods or knowledge [43]. The common core elements widely used to measure SES are education, income (if available), and employment. Other approaches emphasize assets rather than income as a better measure of stable SES. Composite SES measures have been developed [3, 35, 37] but the validity and reliability are often not known [44]. Information on individuals may not be known and SES classification may be based on group level measurements such as neighborhood characteristics [35, 37, 40]. In our study, neighborhood characteristics (e.g. county income) did not add any predictive power of nicotine exposure above the individual level variables identified in the PCA. The wide variety of measures has led to inconsistencies in findings on SES and health disparities [45]. The best measure may depend upon the specific outcome and population under study [46].

The effects of SES on smoking dose or levels of tobacco consumption have been more difficult to understand for a number of reasons. First, characterizing SES is challenging since it is a construct that cannot be directly measured by a single indicator. Income is considered an important component of SES but is often not assessed or difficult to analyze in health research due to nonresponse [47]. Education has been used commonly as a proxy. We employed methods that have been developed to elicit higher response rates including initial broad income categories followed by more specific questions on exact income and additional assurances about the confidentiality of these questions and their importance. While a number of SES indicators were collected including family members’ level of education, and individual questions on pension plans, health insurance and savings account as well as county income data, household income data was the most strongly correlated variable in the SES PCA, and was individually related to key outcomes as well. There are few reports of smoking frequency by income at the individual level. In England, a composite scale of income, employment status and other SES indicators was used to show that lower SES was associated with more cigarettes per day than higher SES but the extent of differences also differed by geography [48]. In Australia, smokers who smoke more than 15 cigarettes per day and have difficulty quitting are more likely to live in disadvantaged geographic areas [17]. In PASS, it was observed that heavier smokers (> 20 CPD) had household incomes nearly $10,000 per year less than lighter smokers. Another challenge in assessing the relationship between SES and levels of smoke exposure is the lack of objective levels of smoke exposure [5]. Misreporting of smoking habits is one concern but variability in smoke intake per cigarette also contributes to significant variability in this measure. There is, for example, over a 20-fold difference in blood levels of cotinine in one pack per day smokers [32]. Cotinine levels also tend to plateau at around 15–20 cigarettes per day, and some smokers who smoke two packs per day have the same cotinine levels as smokers who take fewer than one half pack per day. In the current study we therefore used biochemical levels of nicotine intake to determine levels of exposure in relation to SES. We used not just cotinine but both major nicotine metabolites (cotinine +3HC) as outcome measures, where total metabolites accounts for inter-individual and genetic variation in the metabolism of cotinine to 3HC.

The income and other SES data were analyzed using principal components analysis. In the PCA, the factor loadings for PC1 were all positive, indicating that each variable (household income, home dwelling type [house vs. other] and number of people living in the household), has an increasing relationship with SES. The relationship with number of people living in the household likely reflects the advantages of shared resources, where for example, two household members can carpool to work for the same cost as a single household member [49]. It should be noted that the PASS population is not highly socioeconomically deprived. The mean family household income was $59.2 k. In Pennsylvania, the median income was $55.7 k in 2015. Household number can also be used as a measure of low SES, based on the extent to which children of the opposite sex may have to share a bedroom or adults and children share bedrooms [50]. We did not collect this information but the positive loading for this variable indicates that it likely represents a benefit of shared resources. After adjusting for known predictors of cotinine, the results showed that the SES PC1 was associated with cotinine and TSNM but that the findings varied by the main contributor to PC2 (e.g. occupation category). Lower SES was significantly associated with higher levels of TSNM in the unemployed. Lower SES was associated with higher levels of TSNM in blue-collar workers but the relationship was not statistically significant. Grouping jobs into 3 main employment categories is convenient and provides for statistical power to detect differences but these broad groupings may hide underlying relationships. It is possible that a significant relationship exists in subgroups of blue-collar jobs. The topography data is consistent with these findings where unemployed subjects had significantly higher number of daily puffs compared to white collar workers, and blue-collar workers had intermediate levels between the two other groups. Surprisingly, there was a positive relationship between SES and TSNM in white-collar workers. This relationship may be due compensatory smoking within this group as increasing SES was associated with higher levels of puff volume in white collar workers. This relationship was not found in the two other job groups. It is possible for example, that the higher income white collar earners smoke more intensely due to greater stress than the lower income white collar workers and/or this may occur for one sex but not the other. Further work would be needed to explore this relationship. It should be noted that educational level was not a significant predictor of outcomes using PCA whether scaled as a categorical or continuous variable. This may be because it is correlated with income in our data and likely in general but the unit increase in income explains more variability than education. Additional file 3 shows one of our univariate models of education categories in relation to the tobacco smoke biomarkers. The adjusted R2 values are lower than that for the PCA models.

This study is not the first to indicate a relationship between SES and a biochemical measure of nicotine exposure. Higher levels of education predicted lower serum thiocyanate levels in the 1992 Czech MONICA study, and lower plasma cotinine levels in the 2007 FINRISK study [27, 51]. Lower income was associated with higher cotinine in NHANES and the Health Survey for England [28–30]. Consistent with these findings, we found that our SES PC1 measure was predictive of lower levels of cotinine, 3HC and TSNM. However, this relationship changed after adjustment for significant covariates and further stratification by occupation. It also depended somewhat on the specific exposure measure used (e.g. COT, 3HC, TSNM). The inverse association between SES and biochemical markers was only apparent in the unemployed and blue-collar groups.

As noted, while SES and tobacco outcomes have been studied extensively, less is known about its relationship with degree of tobacco exposure. Similarly, there has been a dearth of research on SES and nicotine dependence. In the International Tobacco Control Four Country Survey (ITC-4; 2002 data), income and education was associated with a lower Heaviness of Smoking Index in three countries. Education but not income was significant for U.S. subjects, although significant differences for income were found when comparing the highest vs. lowest income levels. The current study showed that lower household income was significantly associated with the FTND score, and further that the scores varied by occupation type from 5.1 in the unemployed, to 4.4 in blue-collar workers, and 3.8 in white collar workers. These FTND findings are consistent with the differences in the relationship between SES and the biomarkers between these groups.

There are several limitations of our study including relatively few racial and ethnic minority participants. We had a small proportion of non-whites (12%), which is representative of the population demographics of central Pennsylvania but limits our ability to generalize the findings to a non-white population. Secondly, the design is cross-sectional, and that limits our ability to understand the causal relationships that underlie the statistical associations. Psychosocial stress, less social support, greater exposure to tobacco advertising, and living with another smoker in the household may all contribute to increased tobacco smoke exposure. We cannot conclude, for example, that unemployed or blue collar workers smoke more due to stress or other factors associated with their job status or have more opportunities to smoke.

## Conclusions

The PASS study was specifically designed to include a number of measures of SES, and we used principle components to create a summary indicator of SES. In this population and with regard to smoking exposure as an outcome, the study indicates that important SES measures for smoking are household income, number in household, type of dwelling and occupation. In general, lower SES smokers smoke more frequently, have higher levels of dependence and tobacco smoke exposure.

## Additional files

Additional file 1: Eigenvalues of the Covariance Matrix. Eigenvalues of the covariance matrix for the derived factors. (DOCX 12 kb)

Additional file 2: Eigenvectors – Factor Loadings. Eigenvectors of all factor loadings. (DOCX 13 kb)

Additional file 3: Baseline Model of Education and Nicotine Metabolite Measures. Univariate models of education in relation to the tobacco smoke biomarkers (DOCX 12 kb)

## Abbreviations

3HC: 3-‘hydroxycotine
COT +3HC: Total salivary nicotine metabolites
COT: Cotinine
FTND: Fagerström Test for Nicotine Dependence
NHANES: National Health and Nutrition Examination Surveys
PASS: Pennsylvania Adult Smoking Study
PCA: Principal component analysis
PhenX: Consensus Measures of Phenotypes and Exposures
SES: Socioeconomic status
SPA-M: Smoking Puff Analyzer-Mobile
