# Supplementary material for: Socioeconomic differences in nicotine exposure and dependence in adult daily smokers
Source: BMC Public Health. 2019 Apr 3;19:375. doi: 10.1186/s12889-019-6694-4 (PMC6448228; doi:10.1186/s12889-019-6694-4)
Supplement: Supplementary file 1 — Eigenvalues of the Covariance Matrix. Eigenvalues of the covariance matrix for the derived factors. (DOCX 12 kb) [file 12889_2019_6694_MOESM1_ESM.docx]

**Additional Table 1 - Eigenvalues of the Covariance Matrix**

| **Factor** | **Eigenvalue** | **Difference**  **(in eigen value)** | **Proportion Explained**  **by Eigen value** | **Cumulative**  **Proportion explained** |
| --- | --- | --- | --- | --- |
| **1** | 1.168 | 0.237 | 0.314 | 0.314 |
| **2** | 0.931 | 0.572 | 0.251 | 0.565 |
| **3** | 0.359 | 0.102 | 0.097 | 0.661 |
| **4** | 0.257 | 0.040 | 0.069 | 0.730 |
| **5** | 0.217 | 0.019 | 0.058 | 0.789 |
| **6** | 0.198 | 0.041 | 0.053 | 0.842 |
| **7** | 0.157 | 0.007 | 0.042 | 0.884 |
| **8** | 0.150 | 0.031 | 0.040 | 0.925 |
| **9** | 0.119 | 0.003 | 0.032 | 0.957 |
| **10** | 0.116 | 0.070 | 0.031 | 0.988 |
| **11** | 0.046 |  | 0.012 | 1.000 |
