# Supplementary material for: Socioeconomic differences in nicotine exposure and dependence in adult daily smokers
Source: BMC Public Health. 2019 Apr 3;19:375. doi: 10.1186/s12889-019-6694-4 (PMC6448228; doi:10.1186/s12889-019-6694-4)
Supplement: Supplementary file 2 — Eigenvectors – Factor Loadings. Eigenvectors of all factor loadings. (DOCX 13 kb) [file 12889_2019_6694_MOESM2_ESM.docx]

**Additional Table 2: Eigenvectors – Factor Loadings**

| **Variable \ Component** | **1** | **2** | **3** | **4** | **5** | **6** | **7** | **8** | **9** | **10** | **11** |
| --- | --- | --- | --- | --- | --- | --- | --- | --- | --- | --- | --- |
| **Income**  **std** | 0.779 | 0.548 | 0.131 | -0.035 | -0.200 | -0.055 | -0.073 | 0.127 | -0.092 | 0.024 | -0.003 |
| **House Number**  **std** | 0.568 | -0.794 | -0.137 | -0.011 | 0.023 | 0.088 | 0.010 | 0.066 | -0.121 | 0.023 | 0.017 |
| **High ed (BS or Greater)** | 0.036 | 0.110 | -0.185 | 0.359 | 0.487 | -0.306 | 0.443 | 0.263 | -0.381 | -0.287 | -0.026 |
| **High**  **Job type (White collar)** | 0.074 | 0.164 | -0.563 | -0.023 | 0.086 | 0.214 | 0.056 | -0.298 | 0.028 | 0.038 | 0.710 |
| **Low**  **Job Type (Blue)** | 0.012 | -0.101 | 0.765 | 0.063 | 0.164 | 0.033 | 0.195 | -0.016 | -0.002 | 0.027 | 0.577 |
| **Employed Type** | 0.116 | 0.108 | 0.117 | 0.155 | 0.268 | 0.628 | 0.296 | -0.470 | -0.065 | 0.107 | -0.385 |
| **High**  **County (by income; ≥55.2k vs other)** | 0.002 | -0.046 | -0.015 | 0.911 | -0.325 | -0.007 | -0.148 | -0.045 | 0.166 | 0.094 | 0.051 |
| **Dad education (some college higher vs other)** | 0.012 | 0.058 | -0.046 | 0.062 | 0.511 | -0.034 | -0.284 | 0.265 | 0.061 | 0.758 | -0.011 |
| **Mom education (some college higher vs other)** | 0.078 | 0.031 | 0.055 | 0.083 | 0.438 | 0.240 | -0.588 | 0.095 | 0.242 | -0.565 | 0.012 |
| **Home Type (home vs other)** | 0.205 | -0.026 | -0.029 | -0.031 | 0.203 | -0.422 | 0.282 | -0.269 | 0.758 | -0.019 | -0.101 |
| **Mortgage (Y vs N)** | -0.037 | 0.037 | -0.090 | -0.021 | -0.130 | 0.467 | 0.376 | 0.669 | 0.404 | -0.022 | 0.034 |

Std indicates a standardized variable
