# Supplementary material for: Socioeconomic differences in nicotine exposure and dependence in adult daily smokers
Source: BMC Public Health. 2019 Apr 3;19:375. doi: 10.1186/s12889-019-6694-4 (PMC6448228; doi:10.1186/s12889-019-6694-4)
Supplement: Supplementary file 3 — Baseline Model of Education and Nicotine Metabolite Measures. Univariate models of education in relation to the tobacco smoke biomarkers (DOCX 12 kb) [file 12889_2019_6694_MOESM3_ESM.docx]

**Additional Table 3. Baseline Model of Education and Nicotine Metabolite Measures**

|  | **Cot** | | **3HC** | | **TSNM** | |
| --- | --- | --- | --- | --- | --- | --- |
| **Variables** | Coeff | p-value | Coeff | p-value | Coeff | p-value |
| **Intercept** | 52.28 | 0.1813 | 4.79 | <.0001 | -2.03 | <.0001 |
| **Age** | 4.17 | <.0001 | 0.13 | <.0001 | 0.01 | <.0001 |
| **Race**  Non-White vs White | -37.48 | 0.1493 | -2.29 | 0.0005 | -0.11 | 0.0764 |
| **Sex**  M vs F | 66.61 | <.0001 | 0.35 | 0.3946 | 0.13 | 0.0012 |
| **Ed level**  Less than HS vs BD or Greater  HS/GED vs BD or Greater  Some College vs BD or Greater  Associates vs BD or Greater | 83.27  45.62  68.20  94.77 | 0.0466  0.1239  0.0323  0.0059 | 1.52  0.28  0.25  0.57 | 0.1390  0.6975  0.7460  0.4908 | 0.22  0.14  0.16  0.21 | 0.0243  0.0400  0.0299  0.0075 |
|  |  |  |  |  |  |  |
| **Adj R2** | 0.1126 | | 0.1522 | | 0.1291 | |

HS: High School degree or equivalent. BD: Bachelor’s Degree.
